# Specific cognitive impairment predicts the neuropsychiatric symptoms in patient with mild cognitive impairment

**DOI:** 10.1007/s40520-025-02952-6

**Published:** 2025-03-01

**Authors:** Chenxi Pan, Ningxin Dong, Xiao Yuan, RenRen Li, Jing Ma, Ying Su, Qinghua Wang, Zhilan Tu, Jialin Zheng, Yunxia Li

**Affiliations:** 1 Department of Neurology, Tongji Hospital, School of Medicine, Tongji University, 389 Xincun Road, Putuo District, Shanghai, 200092 China; 2 Department of Medical Imaging, Tongji Hospital, School of Medicine, Tongji University, No.389 Xincun Road, Shanghai, China; 3 Department of Neurology, Shanghai Pudong Hospital, Fudan University Pudong Medical Center, 2800 Gongwei Road, Pudong New District, Shanghai, 201399 China; 4 Shanghai Key Laboratory of Vascular Lesions Regulation and Remodeling, No.2800 Gongwei Road, Shanghai, China

**Keywords:** Neuropsychiatric symptoms, Mild cognitive impairment, Memory, Language

## Abstract

**Background:**

Neuropsychiatric symptoms (NPS) are common in mild cognitive impairment (MCI). However, knowledge is limited about the relationship of NPS, clinical factors, and cognition in MCI.

**Methods:**

A total of 1099 dementia, 1323 MCI and 377 cognitively normal (CN) were selected from the Tongji Cohort Study of Aging. All participants underwent comprehensive clinical and neuropsychological assessment. NPS were evaluated by the Neuropsychiatric Inventory Questionnaire (NPI-Q). Logistic regression analyses were conducted to investigate the relationship between clinical factors, cognition and NPS.

**Results:**

The NPS presented in 56.39% of MCI participants, and the NPI-Q scores of MCI was intermediate between CN and dementia. The most common NPS in MCI were depression (30.76%), anxiety (25.09%), apathy (19.43%), and irritability (12.02%). MCI patients with NPS showed worse performance in global, memory, language, and attention than those without NPS. Additionally, Logistic regression analyses revealed that MCI patients with ischemic heart disease (OR = 1.41; 95%CI 1.050–1.897; *P* = 0.022) were more likely to have NPS, but MCI patients with increased memory domain Z score (OR = 0.847, 95%CI = 0.720–0.996, *p* = 0.044), and language domain Z score (OR = 0.801, 95%CI = 0.682–0.941, *p* = 0.007) were less likely to have NPS.

**Conclusions:**

Neuropsychiatric symptoms occur commonly in MCI participants, and are mainly related to defect of language and memory function. A better understanding of the relationship between specific cognition and NPS may alert clinicians to pay close attention to the NPS in MCI patient, which may need early intervention.

**Supplementary Information:**

The online version contains supplementary material available at 10.1007/s40520-025-02952-6.

## Introduction

Neuropsychiatric symptoms (NPS) refer to non-cognitive, behavioral or psychiatric dysfunctions, including disturbances of mood, behavior, and perception, which are related to a neurocognitive disorder [1]. NPS were once thought to be clinical symptoms restricted to patients with dementia, but now they are considered as a non-cognitive dysfunction that can occur in patients with mild cognitive impairment (MCI), and even in participants without cognitive impairment. The NPS score are significantly different among normal controls, MCI and Alzheimer’s Disease (AD) [2]. But most studies only report the prevalence of NPS in patients with MCI and dementia [3–6], few studies include cognitive normal elderly, MCI and dementia in one setting [7], which are community or nursing-home based, with sample fewer than 900 participants. Researches on the frequency of all NPS in individuals with normal cognition, MCI and dementia are needed, which are in a large clinic-based study.

MCI is regarded as a transitional state between the cognitive changes of normal aging and early dementia, in which patients have cognitive decline complaints but that decline have no relevant impact on the day-living activities [8, 9]. Recently, it has been reported that neuropsychiatric symptoms are common early features of MCI patients [10–12]. These symptoms correlate with greater functional impairments and increase the risk of cognitive deterioration [13–16], creating a considerable burden for patients and their caregivers [17], which implies higher economic and social costs [18]. Thus, the early identification of NPS in MCI is an important avenue to explore early interventions which could prevent cognitive impairment.

Relationships between single neuropsychiatric symptom and MCI have been explored in many studies. A meta-analysis report that depression predominates in MCI [19]. Apathy is also one of the most prevalent behavioral symptoms in MCI [3, 20], and MCI with apathy show poorer performance on decision-making tasks than those without apathy [21]. However, previous studies mainly focus on a single symptom, and do not take the correlations with other NPS into account. In fact, many NPS tend to co-occur, overlap, and even covary [22, 23]. Therefore, these studies may lack the sensitivity to more widespread associations. Neuropsychiatric Inventory (NPI) measure 12 neuropsychiatric symptoms, adjusting each of the symptoms for the other NPS, and allow for the correlations between symptoms. Using NPI, Feldman et al. find MCI patients with NPS have lower score on MMSE and AD Assessment Scale–cognitive subscale (ADAS-cog) than those without NPS [15]. Prior studies on specific cognitive domains and neuropsychiatric symptoms is not completely consistent. Rosenberg et al. find executive dysfunction was associated with greater NPI total severity [24], while Brodaty et al. observe, except executive, cognitive dysfunctions of attention and global cognition were also associated with total NPS [25]. In addition, previous researches on sex differences in NPS report mixed findings. Several studies suggest females showed a greater and wider range of NPS [26, 27], but others don’t find any sex differences in the prevalence and severity of NPS in AD [28, 29]. Furthermore, undiagnosed medical conditions and specific illnesses, such as hyperglycemia, anemia, and thyroid disorders, are associated with NPS in dementia [30, 31]. However, prior studies mainly focus on dementia patients, and findings on MCI are surprisingly rare. Whether clinical, demographic characteristics and specific cognition may play a role in NPS in MCI needs further investigation.

Our study aims to address the above-mentioned gaps in the literature to (1) investigate the frequency of NPI in a memory clinic-based cohort across different stages from normal cognition, MCI to dementia; (2) explore whether there are differences in clinical factors and cognition in MCI patients with NPS or without NPS; and (3) explore the relationship between clinical factors, cognitive function and neuropsychiatric symptoms.

## Materials and methods

### Setting

The Tongji Cohort Study of Aging (TCSA) is a clinic-based study designed to study the neuroimaging changes, disease progression, and neurophysiological mechanism of dementia patients at different disease stages. The TCSA study protocols have been approved by the Medical Ethics Committee of Tongji Hospital Affiliated with Tongji University. After a detailed description of the study, written informed consent have obtained from all participants.

### Participants

Participants aged ≥ 50 years were recruited from Department of Neurology and Memory Clinic of Tongji Hospital from January 2018 to April 2023. All participants underwent the following standard face-to-face evaluations: (1) neurological evaluation and risk factor assessment were performed by a physician, (2) neuropsychological evaluation was interpreted by a neuropsychologist. The neurological evaluation included medical history, pre-existing diseases, medication history, family history and a complete neurological examination. Standardized structured questionnaire, which included risk markers, was obtained via self-report. The final diagnosis of cognitively normal (CN), MCI, or dementia was made by an expert consensus panel consisting of physicians and neuropsychologists based on all available data.

### Neuropsychological evaluation

Neuropsychological evaluation included tests in global cognition and five cognitive domains: (1) global cognition was performed with the Mini Mental State Examination (MMSE) and the Montreal Cognitive Assessment (MoCA); (2) memory was assessed with the Logical Memory subtest (LMT) from Wechsler Memory Scale-Revised, the Hopkins Verbal Learning Test- Revised (HVLT-R, immediate and delayed recall), and the Rey-Osterrieth Complex Figure Test (CFT, delayed recall); (3) attention function was performed with the Trail-Making Test Part A (TMT-A); (4) executive function was assessed with the Trail‐Making Test Part B (TMT-B); (5) language was performed with the Boston Naming Test (BNT) and the Verbal Fluency Test (VFT); (6) visuospatial function was assessed with the CFT (copy). The final decision about impairment in any cognitive domain was determined by an expert consensus panel consisting of physicians and neuropsychologists after they reviewed the results of each participant.

### Measurement of neuropsychiatric symptoms

The neuropsychiatric symptoms are examined using the Neuropsychiatric Inventory Questionnaire (NPI-Q), which is a structured interview with established reliability and validity [32]. The NPI-Q assessed 12 behavioral and psychological domains, which is obtained through the spouse or the caregiver of participant. Every domain is assessed based on its severity and then all domain scores were added together to get a total NPI-Q severity score. Based on the total NPI-Q severity score, MCI patients were divided into a MCI with NPS subgroup (NPI-Q > 0) and a MCI without NPS (NPI-Q = 0) subgroup.

### Statistical analysis

Descriptive statistics were performed with the Statistical Package for the Social Sciences (SPSS) (version 25). Categorical and continuous variables were expressed as frequency and percent or mean and standard deviation (SD), as appropriate. Depending on normal distribution of the continuous variables, Student t-test and Mann-Whitney U test were used to find the difference between the MCI with NPS subgroup and the MCI without NPS subgroup. A Chi-square test was utilized to analyze categorical variables.

To further investigate the associations between the specific cognition domain and NPS in MCI patients, and evaluate if they can improve prediction of NPS, binomial logistic regression models containing clinical and/or cognitive data were carried out. Data associated with outcome at the univariate analysis (p value < 0.10) and variables (e.g. age) based on our own clinical experience were included in models. Model 1 included gender, age, education, BMI, ischemic heart disease, DM, anemia, MoCA; Model 2 added global z score, executive z score, attention z score, language z score, memory z score, visuospatial z score. Variance inflation factor (VIF) was used to test the independence of variables used in different models. No variable used in models had VIF above 2, suggesting the absence of multicollinearity between variables and allowing the models to be used. Statistical significance was set at *p* < 0.05.

## Results

### Descriptive analyses

**Fig. 1:**
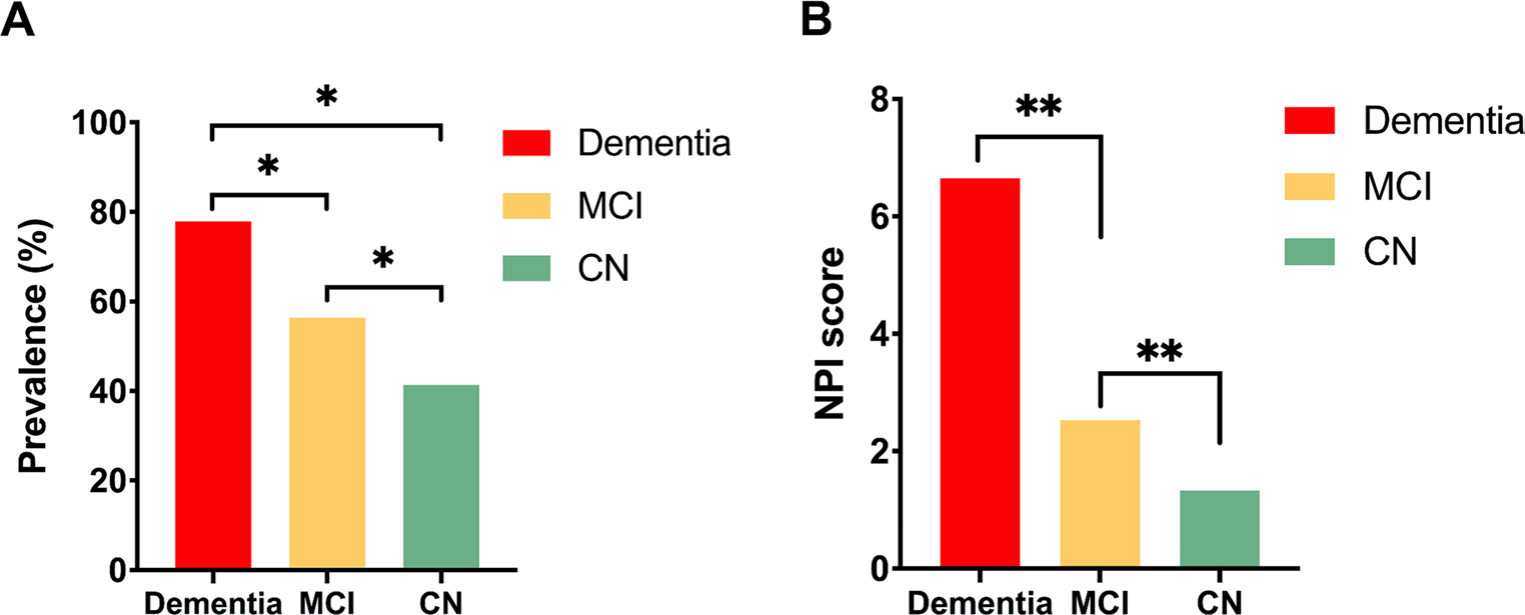
The number and frequency of neuropsychiatric symptoms in three groups. *p < 0.05; **p < 0.001. CN, cognitively normal; MCI, mild cognitive impairment; NPI, neuropsychiatric symptom

The study recruited 3061 participants, of these, 79 participants refused to carry out neuropsychological evaluation, and we excluded 172 individuals missing data on MMSE and/or MOCA (*n* = 166), risk factor assessment (*n* = 6). Therefore, the final sample consisted of 2794 participants. 377 participants of 2794 (13.49%) were CN, 1323 participants (47.35%) were MCI and 1091 participants (39.05%) were dementia. NPS was not only present in dementia, but also in MCI and CN. The frequency of any NPS was 41.38% in CN group, 56.39% in MCI group, 77.88% in dementia group (Fig. 1).

**Fig. 2:**
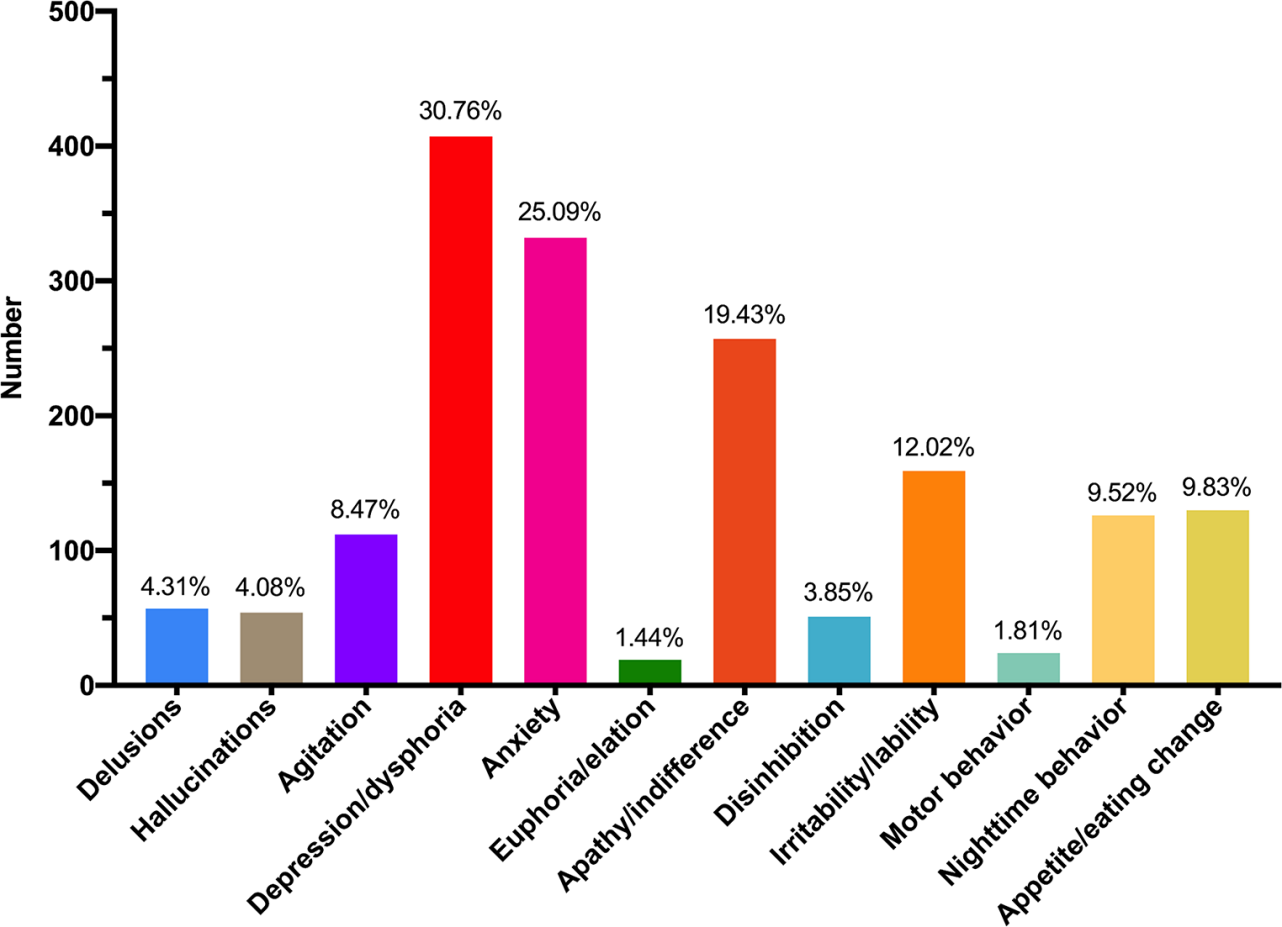
The number and frequency of each neuropsychiatric symptom in MCI group

The total NPI-Q score of MCI group was in the middle of CN and dementia groups. The most frequently endorsed NPS in MCI was depression/dysphoria (30.76%), followed closely by anxiety (25.09%), apathy/indifference (19.43%), and irritability/lability (12.02). The less prevalent NPS was euphoria/elation (1.44%) and motor behavior (1.81%) (Fig. 2). In MCI participants, the neuropsychiatric symptoms were reported in 746/1323 (56.39%) MCI participants (MCI + NPS group), whereas 577/1323 (43.61%) MCI participants did not (MCI-NPS group). The MCI + NPS group had more clinical conditions (e.g. diabetes mellitus and ischemic heart disease) than the MCI-NPS group, but there were no differences in age, education, MCI subtypes, parental history of dementia, smoking and alcohol consumption (Table 1). The MCI + NPS group tended to have more female and lower Body Mass Index (BMI), although this did not reach significant difference, gender and BMI was included as a variable in further logistic regression analyses.

**Table 1:** The demographic and clinical characteristics between two MCI subgroups

	MCI + NPS *n* = 746	MCI only *n* = 577	*P*
Age, years	67.40 ± 8.07	67.73 ± 8.61	0.474
Gender, male (%)	312 (41.82%)	268 (46.45%)	0.093
Education, years	10.39 ± 3.57	10.69 ± 3.60	0.127
BMI	23.36 ± 3.50	23.69 ± 3.36	0.087
MMSE	25.75 ± 2.30	25.68 ± 2.54	0.611
MOCA	18.32 ± 3.91	18.98 ± 3.91	**0.002**
MCI subtypes, n (%) aMCI naMCI	686 (91.96%) 60 (8.04%)	519 (89.95%) 58 (10.05%)	0.204
Smoking, n (%) Never or former smoking Current smoking	526 (70.51%) 220 (29.49%)	418 (72.44%) 159 (27.56%)	0.440
Alcohol consumption, n (%) Never or former drinking Current consumption	670 (89.81%) 76 (10.19%)	515 (89.25%) 62 (10.75%)	0.742
Parental history of dementia, n (%)	125 (16.76%)	88 (15.25%)	0.460
Clinical conditions, n (%)			
Hypertension	386(51.74%)	298 (51.65%)	0.972
Diabetes mellitus	134 (17.96%)	79 (13.69%)	**0.036**
Ischemic heart disease	148 (19.84%)	79 (13.69%)	**0.003**
Hyperlipidemia	255 (34.18%)	189 (32.76%)	0.586
Cerebrovascular disease	165 (22.12%)	115 (19.93%)	0.334
Hyperthyroidism	79 (10.60%)	70 (12.13%)	0.379
Traumatic brain injury	92 (12.33%)	55 (9.53%)	0.108
Anemia	103 (13.81%)	60 (10.40%)	0.061

Data are reported as mean ± standard deviation or n (%). p-values were calculated using the two-sample t test, Mann-Whitney U test, or Chi-square test. The values in bold are statistically significant difference (*p* < 0.05)

*Abbreviations* aMCI, amnestic mild cognitive impairment; BMI, Body Mass Index; CN, cognitively normal; MMSE, Mini Mental State Examination; MoCA, Montreal Cognitive Assessment; MCI, mild cognitive impairment; MCI + NPS, mild cognitive impairment with neuropsychiatric symptom; MCI only, mild cognitive impairment without neuropsychiatric symptom; naMCI, nonamnestic mild cognitive impairment

### Logistic regression

The MCI + NPS group scored significantly lower on MoCA than the MCI-NPS group (*p* < 0.002, Table 1). Moreover, the MCI + NPS group performed significantly worse on HVLT-immediate recall, HVLT-delayed recall, CFT, TMT-A, VFT, and BNT (*p* < 0.05, Table 2). In logistic regression analysis, model 1 revealed that ischemic heart disease (OR = 1.539, 95%CI = 1.141–2.077, *p* = 0.005) and MoCA (OR = 0.957, 95%CI = 0.930–0.984, *p* = 0.002) was significantly associated with presence of NPS in MCI patients (Table 3). In order to explore whether specific cognitive domain was related to the NPS in MCI, we use a battery of neuropsychological tests that assess five cognitive domains. For analysis purposes, we transformed the scores of cognitive tests into Z scores, and then individual neuropsychological test Z scores were averaged to calculate a domain Z score and a global Z score. Model 2 revealed that MCI patients with ischemic heart disease (OR = 1.544, 95%CI = 1.145–2.082, *p* = 0.004) were more likely to have neuropsychiatric symptoms, but MCI patients with increased memory domain Z score (OR = 0.847, 95%CI = 0.720–0.996, *p* = 0.044), and language domain Z score (OR = 0.801, 95%CI = 0.682–0.941, *p* = 0.007) was less likely to have NPS (Table 3).

**Table 2:** Cognitive assessments of MCI participants with and without neuropsychiatric symptoms

	MCI + NPS *n* = 746	MCI only *n* = 577	*p*
**Attention**			
TMT-A (s)	70.35 ± 25.52	65.60 ± 23.26	**0.000**
***Executive***			
TMT-B (s)	166.34 ± 51.27	165.22 ± 50.61	0.691
***Memory***			
HVLT-immediate recall	5.56 + 1.53	5.76 + 1.53	**0.02**
HVLT-delayed recall	3.68 ± 2.90	4.13 ± 2.97	**0.005**
CFT-recall	10.48 ± 7.68	11.56 ± 7.82	**0.012**
LMT	6.57 ± 2.37	6.83 ± 2.51	0.055
***Visuospatial function***			
CFT-copy	31.71 ± 6.09	31.75 ± 5.89	0.961
***Language***			
VFT	12.25 ± 3.55	12.85 ± 3.67	**0.003**
BNT	20.41 ± 4.07	20.93 ± 3.93	**0.020**

Raw scores of neuropsychological tests were presented as mean ± standard deviation. p-values were calculated using the two-sample t test, Mann-Whitney U test. The values in bold are statistically significant difference (*p* < 0.05)

*Abbreviations* BNT, Boston Naming Test; CFT, Rey-Osterrieth Complex Figure Test; DST, Digit Span Backward Test; HVLT, Hopkins verbal learning test; LMT, Logical Memory Test; MCI + NPS, mild cognitive impairment with neuropsychiatric symptom; MCI only, mild cognitive impairment without neuropsychiatric symptom; s, second; TMT-A, Trail Making Test A; TMT-B, Trail Making Test B; VFT, Verbal Fluency Test

**Table 3:** Multivariable regression analysis of neuropsychiatric symptoms with clinical factors and cognitive function in MCI patients

	B	*p*	EXP (𝛽)	95% CI
Model 1				
Ischemic heart disease	0.431	0.005	1.539	1.141–2.077
MoCA	-0.044	0.002	0.957	0.930–0.984
Model 2				
Ischemic heart disease	0.458	0.003	1.581	1.170–2.136
Memory z score	-0.166	0.044	0.847	0.720–0.996
Language z score	-0.222	0.007	0.801	0.682–0.941

Model 1: Gender, Age, Education, BMI, Ischemic heart disease, DM, Anemia, MoCA

Model 2: Gender, Age, Education, BMI, Ischemic heart disease, DM, Anemia, Global z score, Executive z score, Attention z score, Language z score, Memory z score, Visuospatial z score

## Discussion

Here we investigate the frequency of NPS in participants of different stage of cognition in a memory clinic-based cohort, then explore the relationship of neuropsychiatric symptoms, clinical factors, and cognition in MCI. As hypothesized, neuropsychiatric symptoms occur commonly in MCI patients, the presence of NPS in MCI patients are associated with the decline of memory and language, as well as the presence of ischemic heart disease. In addition, the MoCA score is also associated with the presence of NPS in MCI patients.

Our findings suggest that NPS present in participants of different stage of cognition, and is highly frequent in both mild cognitive impairment and dementia. This is generally in line with previous results [4, 14], but these studies, based on population or community-dwelling, reported the frequencies of NPS in MCI (43%, 47%) and dementia (75%, 66.1%) that are lower than ours. The frequencies of NPS in clinic-based studies are higher than that of community-based studies [22], which may due to selection bias, for the NPS of participants who come to hospital may be more severe than those in community. Few studies also explore the NPS in cognitively unimpaired control group [7, 33]. However, the sample of these two studies is smaller (*n* = 856, *n* = 780) than ours (*n* = 2794). A review point that the frequency of neuropsychiatric symptoms largely depends on the sample and the type of setting [34]. On this note, the frequency of NPS in normal cognition group of our study (41.38%) is higher than previous studies (12%, 3%).

The result is plausible that ischemic heart disease is positively associated with the NPS in MCI. The possible mechanisms that link NPS with cognitive dysfunction is that they shared risk factor or confounding [35]. Previous researches have provided evidence linking cognitive decline with coronary heart disease [36–38]. In addition, MCI group with both neuropsychiatric and functional features is more likely to have probable cerebrovascular disease [39]. Moreover, brain autopsies confirm the presence of a mixed pathology consisting of both Alzheimer’s and vascular diseases in a MCI group with neuropsychiatric disturbance, which exhibits greater association with cardiovascular comorbidity [40]. Thus, although there is no causal relationship between neuropsychiatric symptoms and MCI, there is a third factor that causes MCI and NPS to co-occur, such as vascular disease [41].

We discover that the MoCA of the MCI with NPS group is lower than that of the MCI without NPS group, which is consistent with previous study [15]. In further cognitive domain analysis, the MCI with NPS group perform worse on attention, memory and language function, and Brodaty et al. report similarly result [25]. On examining associations between NPS and specific cognitive domain, we find the decline of memory and language z score were risk factors of the presence of NPS in MCI. In this regard, the decline in memory and language function may alerts clinicians to pay attention to the neuropsychiatric symptoms of MCI patient in time. But future longitudinal researches are needed to investigate whether specific cognitive domains are risk factor of NPS.

The main strengths of our study are population setting of the study and its size. In addition, we record the presence or absence of neuropsychiatric symptoms, as well as their severity. Lastly, interviews with both participants and their caregivers are used in our evaluation, which allowed a wide range of demographic and clinical factors to be explored. Nevertheless, some limitations of our research have to be considered. First, our study is a cross-sectional study which do not allow us to attribute a causal relationship between NPS and specific cognitive domain. Second, our study doesn’t examine the dynamic change between NPS and cognitive decline over time. Further longitudinal studies with longer follow-up are needed. Third, previous literature suggests the diversity of executive functions, and recommended that multiple tests should be used for each executive function subcomponent [42]. Therefore, it is not ideal for us to use the TMT-B (measuring cognitive flexibility) as the only assessment of executive function. More neuropsychological tests on executive function, such as Clock Drawing Test (CDT) and Stroop Color Word Test (SCWT), are needed in future studies.

In conclusion, neuropsychiatric symptoms occur commonly across different stages from normal cognition, MCI to dementia. The frequency of NPS and the NPI score in MCI group is in the middle of CN and dementia groups. Compared with those without NPS, the MCI patients with NPS perform worse in cognition, especially in global, attention, memory, and language function. Ischemic heart disease, dysfunction of memory and language function are risk factors of the presence of NPS in MCI patients. Larger longitudinal studies are essential for examining the relationship between NPS and specific cognitive domain in future.

## Electronic supplementary material

Below is the link to the electronic supplementary material.

Supplementary Material 1

## Data Availability

No datasets were generated or analysed during the current study.
