# Supplementary material for: Specific cognitive impairment predicts the neuropsychiatric symptoms in patient with mild cognitive impairment
Source: Aging Clin Exp Res. 2025 Mar 1;37(1):60. doi: 10.1007/s40520-025-02952-6 (PMC11870941; doi:10.1007/s40520-025-02952-6)
Supplement: Supplementary file 1 — Supplementary Material 1 [file 40520_2025_2952_MOESM1_ESM.docx]

|  | **MCI+NPS**  n=746 | **MCI only**  n=577 | **p** | **Bootstrap p** |
| --- | --- | --- | --- | --- |
| Age, years | 67.40 (66.84 - 68.03) | 67.73 (67.05 - 68.45) | 0.474 | 0.484 |
| Education, years | 10.39 (10.13 - 10.64) | 10.69 (10.40 - 10.99) | 0.127 | 0.117 |
| BMI | 23.36 (23.13 – 23.60) | 23.69 (23.41 – 23.96) | 0.087 | 0.077 |
| MMSE | 25.75 (25.58 – 25.91) | 25.68 (25.48 – 25.90) | 0.611 | 0.616 |
| MoCA | 18.32 (18.03 - 18.60) | 18.98 (18.67 – 19.30) | **0.002** | **0.002** |

**Table S1.** The demographic and clinical characteristics between two MCI subgroups.

Data are reported as mean (95% confidence interval)

p values were calculated using the two-sample t test.

Bootstrapping was used to obtain p values and 95% bootstrap confidence intervals for the parameter mean.

**Table S2**. Multivariable regression analysis of neuropsychiatric symptoms with clinical factors and cognitive function in MCI patients

|  | B | p | EXP (𝛽) | 95% CI |
| --- | --- | --- | --- | --- |
| Model 1 |  |  |  |  |
| Ischemic heart disease | 0.438 | 0.004 | 1.550 | 1.150 - 2.090 |
| MoCA | -0.043 | 0.003 | 0.958 | 0.931 - 0.985 |
| Model 2 |  |  |  |  |
| Ischemic heart disease | 0.465 | 0.002 | 1.591 | 1.178 - 2.149 |
| Memory z score | -0.167 | 0.041 | 0.846 | 0.720 - 0.993 |
| Language z score | -0.228 | 0.005 | 0.796 | 0.679 - 0.935 |
| Model 3 |  |  |  |  |
| Ischemic heart disease | 0.431 | 0.005 | 1.539 | 1.141 - 2.077 |
| MoCA | -0.044 | 0.002 | 0.957 | 0.930 - 0.984 |
| Model 4 |  |  |  |  |
| Ischemic heart disease | 0.458 | 0.003 | 1.581 | 1.170 - 2.136 |
| Memory z score | -0.166 | 0.044 | 0.847 | 0.720 - 0.996 |
| Language z score | -0.222 | 0.007 | 0.801 | 0.682 - 0.941 |

Model 1: Age, Education, Ischemic heart disease, Diabetes mellitus, Anemia, Traumatic brain injury, MCI subtypes, MoCA.

Model 2: Age, Education, Ischemic heart disease, Diabetes mellitus, Anemia, Traumatic brain injury, MCI subtypes, Global z score, Executive z score, Attention z score, Language z score, Memory z score, Visuospatial z score.

Model 3: Gender, Age, Education, BMI, Ischemic heart disease, Diabetes mellitus, Anemia, Traumatic brain injury, MCI subtypes, MoCA.

Model 4: Gender, Age, Education, BMI, Ischemic heart disease, Diabetes mellitus, Anemia, Traumatic brain injury, MCI subtypes, Global z score, Executive z score, Attention z score, Language z score, Memory z score, Visuospatial z score.

**Table S3**. Cognitive domains of MCI participants with and without one neuropsychiatric symptom.

|  | **MCI with** | **MCI without** | **p** |
| --- | --- | --- | --- |
| **Delusions, n** | **n = 57** | **n = 1266** |  |
| Memory z score | -0.23 ± 0.84 | 0.01 ± 0.73 | 0.014 |
| Language z score | -0.35 ±0.76 | 0.02 ± 0.74 | 0.000 |
| Attention z score | -0.29 ± 0.83 | 0.01 ± 1.01 | 0.028 |
| Executive z score | -0.34 ± 0.85 | 0.01 ± 0.99 | 0.009 |
| Visuospatial z score | -0.32 ±1.17 | 0.01 ± 0.99 | 0.013 |
| **Agitation, n** | **n = 112** | **n = 1211** |  |
| Memory z score | -0.15 ± 0.79 | 0.01 ± 0.73 | 0.020 |
| Language z score | -0.15 ± 0.81 | 0.01 ± 0.73 | 0.026 |
| Executive z score | -0.29 ± 0.96 | 0.02 ± 0.99 | 0.001 |
| **Depression/dysphoria, n** | **n = 407** | **n = 916** |  |
| Memory z score | -0.07 ± 0.72 | 0.03 ± 0.74 | 0.029 |
| Language z score | -0.12 ± 0.76 | 0.05 ± 073 | 0.000 |
| **Anxiety, n** | **n = 332** | **n = 991** |  |
| Memory z score | -0.07 ± 0.71 | 0.02 ± 0.74 | 0.041 |
| Language z score | -0.08 ± 0.75 | 0.03 ± 0.74 | 0.029 |
| **Euphoria/elation, n** | **n = 19** | **n = 1304** |  |
| Language z score | -0.48 ± 0.78 | 0.01 ± 0.74 | 0.004 |
| Executive z score | -0.50 ±0.87 | 0.00 ± 0.99 | 0.030 |
| Visuospatial z score | -0.56 ± 1.24 | 0.01 ± 0.99 | 0.014 |
| **Apathy/indifference, n** | **n = 257** | **n = 1066** |  |
| Memory z score | -0.12 ± 0.74 | 0.03 ± 0.73 | 0.003 |
| Language z score | -0.12 ± 0.75 | 0.03 ± 0.74 | 0.004 |
| Attention z score | -0.05 ± 1.04 | 0.00 ± 0.98 | 0.013 |
| **Motor behavior, n** | **n = 24** | **n = 1299** |  |
| Executive z score | -0.41 ± 0.82 | 0.00 ± 0.99 | 0.046 |
| **Nighttime behavior, n** | **n = 126** | **n = 1197** |  |
| Language z score | -0.13 ± 0.78 | 0.01 ± 0.74 | 0.044 |

Data are reported as mean ± standard deviation.

p values were calculated using the two-sample t test.
